# Plasmablastic lymphoma of the maxillary sinus in an HIV-negative patient: a case report and literature review

**DOI:** 10.1186/2193-1801-2-142

**Published:** 2013-04-03

**Authors:** Christine Saraceni, Nicole Agostino, Dennis B Cornfield, Ranju Gupta

**Affiliations:** 1 Department of Internal Medicine, Lehigh Valley Health Network, 1255 S Cedar Crest Blvd Suite 3200, Allentown, PA 18104 USA; 2 Section of Hematology-Oncology, Lehigh Valley Health Network, 1240 South Cedar Crest Blvd Suite 103, Allentown, PA18103 USA; 3 Department of Pathology, Section of Hematopathology and Clinical Laboratory Medicine, Health Network Laboratories/ Lehigh Valley Health Network, 1200 South Cedar Crest Blvd, Allentown, PA18103 USA; 4 Section of Hematology-Oncology, Lehigh Valley Health Network, 1240 South Cedar Crest Blvd Suite 103, Allentown, PA18103 USA

**Keywords:** Plasmablastic lymphoma, PBL, HIV-negative, Maxillary sinus

## Abstract

Plasmablastic lymphoma (PBL) is a rare and aggressive variant of diffuse large B cell lymphoma. The prognosis of PBL patients is poor. The majority of patients succumb to a fulminant disease course, with most dying in the first year after diagnosis. The small number of HIV-negative PBL cases reported in the literature to date is composed of single case reports and small case series. Consequently, the natural history of the disease in HIV-negative individuals and the optimum treatment are not well characterized. Intensive induction chemotherapy has been associated with marked improved overall survival. However the optimal regimen has not been defined. We describe the third case of PBL of the maxillary sinus which occurred in a 24-year old HIV-negative man. We outline the clinicopathological features and report success using a hyper-CVAD regimen with 6 cycles and consolidation radiation therapy yielding a complete remission of four years.

## Introduction

Plasmablastic lymphoma (PBL) is a recently recognized aggressive non-Hodgkin’s B-cell lymphoma which occurs predominantly in HIV seropositive individuals and shows a predilection for the oral cavity. Overall, PBL is associated with early dissemination, poor response to therapy and limited survival. To date, treatment responses are usually partial and temporary. Since the first description of PBL in 1997 (Delecluse et al. 1997), the treatment of PBL in HIV-positive patients has been enhanced with the addition of highly active antiretroviral therapy (ART) (Guan et al. 2011; Castillo et al. 2010). However, a small retrospective analysis (Castillo et al. 2012) found that HIV-associated PBL has a poor overall prognosis which is not impacted favorably by more intensive chemotherapeutic regimens in the ART era.

We report an unusual case of plasmablastic lymphoma (PBL) of the maxillary sinus in a young HIV-negative man. To our knowledge this is the third reported case (Nguyen et al. 2003; Colomo et al. 2004) of this entity originating in the maxillary sinus. There have been 79 previously reported cases of HIV-negative PBL, with a majority of these cases arising in the post-transplant setting or immunosuppressed state. Only a small subset of reported cases have occurred in immunocompetent patients. (Delecluse et al. 1997; Nguyen et al. 2003; Colomo et al. 2004; Scheper et al. 2005; Takahashi et al. 2009; Thakral et al. 2009; Teruya-Feldstein et al. 2004; Kim et al. 2009; Cha et al. 2010; Kravetz et al. 2006; Masgala et al. 2007;Lin et al. 2004; Khurana & Jaipota 2010; Pruneri et al. 1998; Lee et al. 2006; Gogia & Bakhshi 2010; Lipstein et al. 2010; Mihaljevic et al. 2011; Guan et al. 2011; Brahmania et al. 2011; Mondal et al. 2011; Mansoor et al. 2012) Table 1. A standardized, optimal chemotherapeutic regime for PBL is yet undefined. To date, initial therapy has included lymphoma-specific multi-agent systemic chemotherapy with or without consolidation radiation and hematopoietic stem cell transplantation. The present case demonstrates a durable clinical, pathologic and radiographic remission of PBL following aggressive chemotherapy with the MD Anderson hyper-CVAD regimen (Kantarjian et al. 2000), and consolidation radiation therapy yielding a complete remission of four years. This report highlights a feasible treatment approach in HIV-negative PBL patients and contributes to the small but increasing body of reported cases.

**Table 1:** **Reported plasmablastic lymphoma cases in HIV seronegative, immunocompetent patients with outcomes**

Report	Demographics	Location	EBV +	Treatment regimen	Prognosis
Delecluse et al. 1997	75 F	Gingiva	UNK	RT (UNK)	↓ 3 mo *
Pruneri et al. 1998	53 F	Gastric	UNK	PROMACE / cytaBOM x 6 cycles	↑19 mo
Nguyen et al. 2003	42 M	Nasal cavity Sinuses	(+)	Hyper-CVAD → RT (40 Gy)	↑ 6 mo
Colomo et al. 2004	56 F	Oral Mucosa	(−)	UNK	UNK
	86 F	Maxillary Sinus	(+)	UNK	↓ 4 mo
	82 M	Lymph Node	(+)	UNK	UNK
Lin et al. 2004	82 M	Cervical LN	(+)	CHOP x 6 cycles	UNK
Teruya-Feldstein et al. 2004	56 M	Sigmoid colon	(−)	CODOX/M-IVAC	↓ 3 mo
	23 M	Neck mass, sinus	UNK	Hyper-CVAD,	↓12 mo
	49 M	Bone		PBSCT	↓ 14 mo
	61 M	Liver, lung	(−)	CHOP x 6 cycles	↓ 12 mo
			(+)	CODOX/M-IVAC	
Scheper et al. 2005	49 M	Mandible	(+)	UNK	UNK
Kravetz et al. 2006	66 M	Upper Extremity	(+)	Hyper-CVAD	↑ 15 mo
Lee et al. 2006	66 M	Gingival Mass	(−)	Chemotherapy → RT (UNK)	↓ 8 mo
Masgala et al. 2007	67 F	Visceral cranium, cervix, thorax	(−)	Cisplatin, 5-FU, leukovorin x 6 cycles	↓ 23 mo
				→ CHOP x 6 cycles	
				→ CHOP-bleomycin	
				→ RT (2000 Gy)	
Kim et al. 2009	67 M	Terminal ileum	(−)	Surgery	↓ 3 mo
	66 M	Oral cavity	(−)	Chemotherapy →	↓ 8 mo
	8 M	Tonsil	(−)	RT (UNK)	↑ 36 mo
	72 F	Paranasal sinus	(+)	Chemotherapy	↑ 6 mo
	61 M	Stomach	(−)	(UNK)	↓ 3 mo
	13 M	Meninges	(−)	Chemotherapy (UNK) Surgery	↓ 7 mo
				Chemotherapy → RT (UNK)	
Takahashi et al. 2009	76 M	Retroperitoneum	(+)	Prednisolone	↓ 35 days
Thakral et al. 2009	84 F	Psoas muscle	(−)	RT (UNK)	↓ 1 mo
Cha et al. 2010	60 M	Jejunum	(−)	CHOP x 6 cycles	↑ 24 mo
				→ ESHAP salvage	
				→ RT (UNK)	
Gogia and Bakhshi 2010	2 F	Jaw - Mandible	UNK	Chemotherapy (UNK)	↓ sepsis
				→ RT (4 Gy)	
Khurana and Jaipota 2010	55 M	Cervical LN	UNK	CHOP	UNK
Lipstein et al. 2010	68 M	Cervical LN	(−)	R-CHOP, DICE, R-CBortP	↓ 1 mo
Mihaljevic et al. 2011	60 M	Gastric	(−)	CHOP	↓ 1 mo
Guan et al. 2011	58 M	Posterior teeth mucosa	(−)	Chemotherapy → XRT	↓ 1 mo
Brahmania et al. 2011	59 M	Ano-rectal junction	(−)	CHOP x 3 cycles → XRT	↑ 5 years
Mondal et al. 2011	47 F	Humerus	UNK	CHOP x 3 cycles	↑ 12 mo
Mansoor et al. 2012	77 F	Cecal/Lung/LN	(−)	High dose steroids	↓ 3 weeks
Present Case 2012	24 M	Maxillary Sinus	(+)	Hyper-CVAD → RT (45 Gy)	↑ 4 years

RT – radiotherapy, UNK – unknown, ↑ alive, ↓ died of disease, EBV – Epstein-Barr virus, HIV – Human Immunodeficiency Virus, M – male, F – female, y – years old, LN – lymph nodes, * dead of unrelated causes, Gy – gray (unit), PBSCT – peripheral blood stem cell transplant.

## Case report

A 24- year old Hispanic man presented with symptoms of chronic sinusitis for two months. He complained of nasal congestion, left-sided facial asymmetry, pain in the left cheek region as well as numbness around the left nostril and left side of the upper lip. Additional constitutional complaints included low grade fever and intermittent night sweats in the 1 – 2 months prior to presentation. He underwent two courses of antibiotics with minimal response. His medical history was unremarkable, including no prior history of sexually transmitted diseases, HIV infection or immunosuppressive conditions.

On physical examination, the patient’s face was grossly asymmetric with left cheek swelling that crowded the left eye. Extraocular muscles and pupillary responses were intact bilaterally. Intraoral examination showed protrusion of the mucosal aspect of the left cheek. The mass was abutting the left nostril. A one centimeter left submandibular lymph node was palpable.

**Figure 1:**
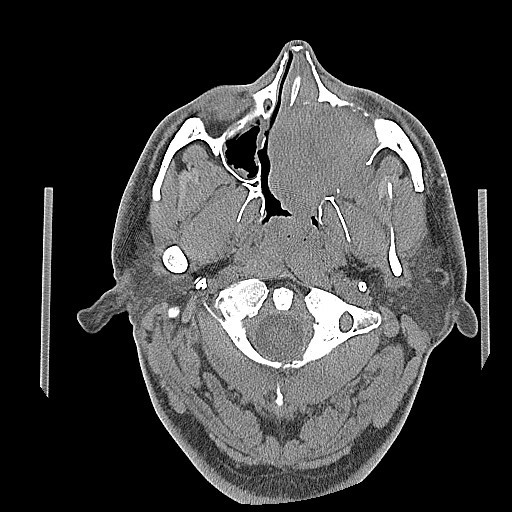
**CT image of the brain, axial view – There is a large expansile soft tissue mass centered within the left maxillary sinus with extensive osseous dehiscence.** Tumor extends into the left orbital floor, nasal cavity, left nasopharynx, left pterygopalatine fossa, left premaxillary space, and left infratemporal fossa.

**Figure 2:**
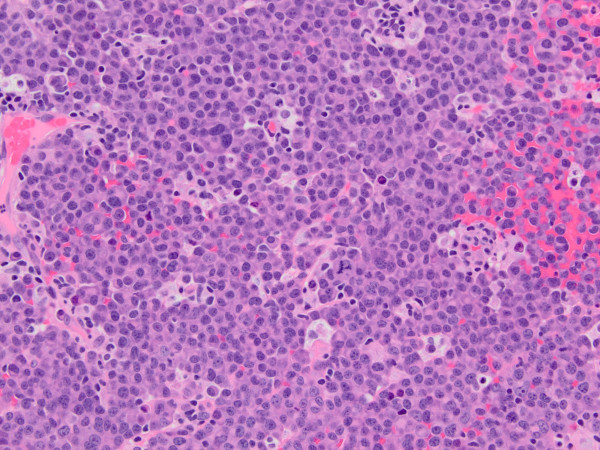
**H&E , (hematoxylin and eosin), large sheets of mostly large plasmacytoid appearing mononuclear cells with moderately dispersed nuclear chromatin and one to several small nucleoli are present.**

**Figure 3:**
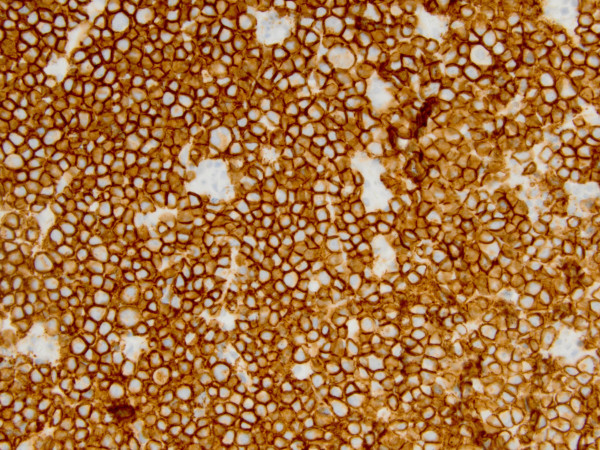
**CD138, immunohistochemical stain demonstrates plasmacytic differentiation.**

**Figure 4:**
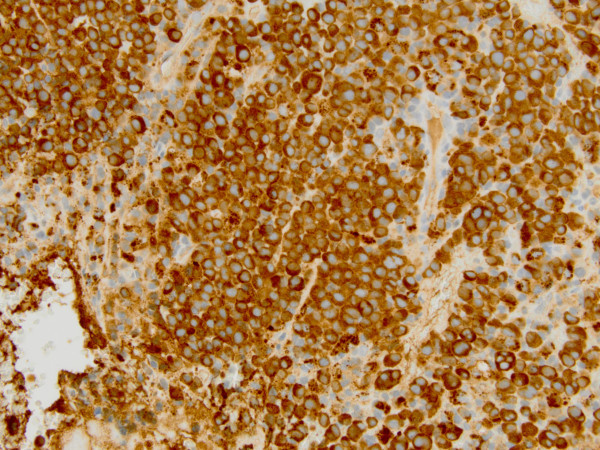
**Lambda, light chain immunohistological stain shows positivity in neoplastic cells.**

**Figure 5:**
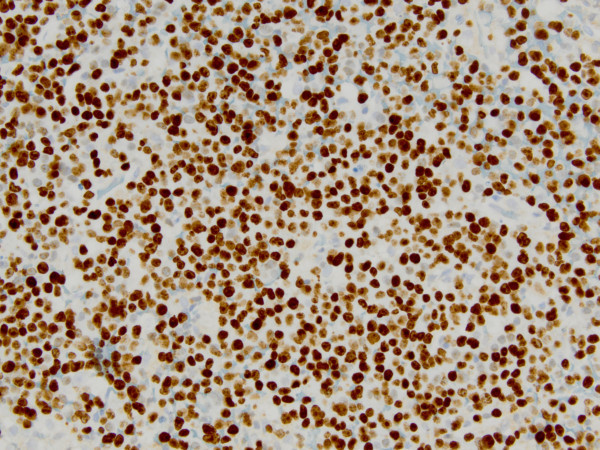
**Ki-67, immunohistochemical stain shows a high proliferative rate.**

**Figure 6:**
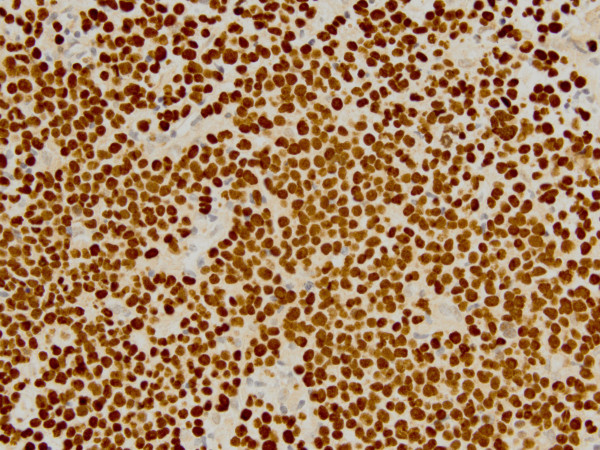
**EBER in situ hybridization shows, extensive positivity.**

**Figure 7:**
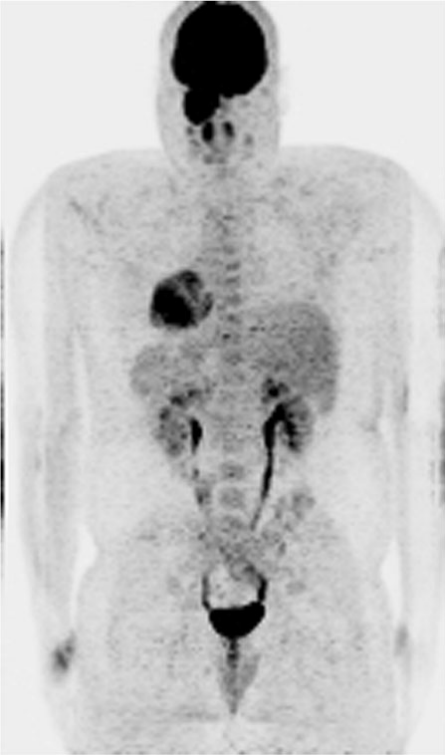
**Whole Body PET/CT (a) Diagnostic Staging PET/CT – There is a hypermetabolic mass centered in the left maxillary sinus. The left cervical lymph nodes between the sternocleomastoid muscle and internal jugular vein as well as subcentimeter level 2 lymph nodes are also hypermetabolic.**

Computed tomography (CT) scan of the head and neck revealed a 5.3 x 5.0 cm left maxillary sinus mass, involving the nasal septum and extending through the medial maxillary sinus wall, into the left nasal canal (Figure 1). The mass also invaded the inferior orbital rim and abutted the inferior rectus muscle. Biopsy of the mass revealed a monotonous, highly proliferative sheet of mononuclear cells (Figure 2). Neoplastic cells were strongly positive for CD 138 (Figure 3), lambda light chain (Figure 4) and Ki-67 [90% expression, Figure 5). In situ hybridization revealed extensive positivity for Epstein-Barr virus-encoded small RNA (EBER) [Leica, Buffalo Grove, IL] (Figure 6). All other negative markers included CD20, CD 56, LCA (CD45), CD3, CD10, kappa light chain, BCL 1, BCL 2, BCL 6, and EBV Latent Membrane Protein. Further staging workup included a bone marrow biopsy and aspirate which did not show any evidence of plasma cell dyscrasia or plasmablastic lymphoma. PET/CT scan showed the 5.3 × 5 cm hypermetabolic mass in the left maxillary sinus, a 14 × 6 mm cervical lymph node between the sternocleidomastoid muscle and internal jugular vein and bilateral sub-centimeter level II lymph nodes (Figure 7). No other evidence of metastatic disease was present. Since the mass involved maxillary sinus, a diagnostic lumbar puncture was also performed which was negative by cytology and flow cytometry for involvement by the lymphoma. Laboratory studies showed normal chemistries, mildly elevated serum lactate dehydrogenase at 217 IU/L [normal <190 IU/L] Beta-2 microglobulin was normal at 1.4 mg/L [range 0.7 – 3.4 mg/L]. Serum and urine protein electrophoresis and immunofixation showed no evidence of monoclonal gammopathy. Enzyme-linked immunosorbent assay /Western Blot for HIV 1 and 2 were negative. Hepatitis A, B, and C serologies, polymerase chain reaction for herpes simplex virus 1 and 2 and human herpesvirus 8 were negative. The lymphoma was staged as IIE and the IPI Score was 0.

**Figure 8:**
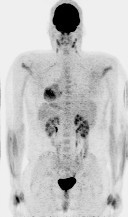
**Whole Body PET/CT (b) Post-treatment – There is no significant FDG uptake seen within the left maxillary sinus, consistent with complete metabolic response to therapy.** There are no pathologically enhancing cervical, axillary, mediastinal or hilar lymph nodes or other sites of metabolically active tumor.

The patient received 6 cycles of chemotherapy with hyper-CVAD involving high doses of cyclophosphamide, vincristine, doxorubicin and prednisone (odd cycles x3), alternating with high doses of methotrexate and cytosine arabinoside (even cycles x 3). He received intrathecal methotrexate and cytosine arabinoside with each of the six cycles of chemotherapy and tolerated treatment well**.** Toxicities during treatment included febrile neutropenia with coagulase negative staphylococcus ventriculitis requiring Ommaya shunt removal and prolonged intravenous and intrathecal vancomycin after cycle 2 as well as Herpes zoster and *Giardia lamblia* infections after cycle 6. A repeat biopsy from the maxillary sinus showed the patient to be in complete pathologic remission confirming negative radiographic findings (Figure 8). He also underwent post-chemotherapy consolidation radiation by intensity-modulated radiation therapy (IMRT) for a total dose of 45 Gy. He patient is still alive and well 4 years after the initial diagnosis with no evidence of recurrence.

## Discussion

Plasmablastic lymphoma is a rare and rapidly progressive variety of diffuse large B-cell lymphoma that was originally reported exclusively in the jaw and oral mucosa of male-predominant HIV-positive patients (Delecluse et al. 1997; Colomo et al. 2004; Yotsumoto et al. 2009). Its hallmarks include extensive local invasion, rapid dissemination and recalcitrance to treatment (Colomo et al. 2004; Scheper et al. 2005; Valenzuela et al. 2008). PBL is overwhelmingly associated with immunodeficiency states particularly precedent HIV infection. A substantial minority of cases occur in HIV-negative patients following solid organ transplantation or immunosuppressive therapy (Colomo et al. 2004; Takahashi et al. 2009; Raviele et al. 2009). Epstein-Barr virus (EBV) infection has been observed in 74% of published PBL cases (Castillo et al. 2008) and may be involved in the pathogenesis of PBL (Raviele et al. 2009). The role of Human Herpes Virus 8 (HHV8) in the pathogenesis of PBL is uncertain (Castillo et al. 2008; Vega et al. 2005). Overall, prognosis in PBL of dismal, with typically median survival of less than one year, particularly in patients with extra-nodal disease (Thakral et al. 2009; Teruya-Feldstein et al. 2004; Raviele et al. 2009). The advent of highly active antiretroviral therapy (ART) has favorably impacted survival in HIV-positive patients in some studies (Teruya-Feldstein et al. 2004; Valenzuela et al. 2008; Raviele et al. 2009). Contrarily, other studies report poor progression-free survival and overall survival despite intensive chemotherapeutic regimens and ART (Castillo et al. 2012). Recently, it has been suggested that HIV-negative patients with PBL have a worse prognosis and a reduced response to chemotherapy than their HIV-positive counterparts on highly active antiretroviral therapy (Colomo et al. 2004; Liu et al. 2011). Nevertheless, the highly aggressive and metastatic nature of PBL along with poor treatment response renders long term survival disappointing (Valenzuela et al. 2008).

Plasmablastic lymphoma is characterized by a terminally differentiated B-cell immunophenotype with minimal or absent expression of leukocyte common antigen (CD45), epithelial markers and B-cell antigens (CD20 and CD79a) but is invariably immunoreactive for well-differentiated plasma cell markers such as CD138 and frequently exhibits monotypic light chain expression (Thakral et al. 2009; Teruya-Feldstein et al. 2004; Raviele et al. 2009). PBL shares many cytomorphologic and immunophenotypic features with plasmablastic plasma cell myeloma (Vega et al. 2005). EBER positivity favors the diagnosis of PBL (Vega et al. 2005; Ramalingam et al. 2008). CD56 expression in diffuse large B cell lymphoma is rare. However, its expression has been reported in PBL (Vega et al. 2005). Histopathologically, PBL shows a diffuse pattern with a high mitotic index (Colomo et al. 2004; Scheper et al. 2005).

Unified treatment guidelines for plasmablastic lymphoma have not been established and treatment regimes have been largely varied and based upon physician discretion. To date, the mainstay of treatment consists primarily of chemotherapy, with the occasional use radiotherapy. The present case is exceptional in that there are only a few other instances in the literature (Kim et al. 2009; Liu et al. 2011) where an HIV-negative, immunocompetent patient with PBL has survived to the 4 year mark. Treatment regimes for immunocompetent patients are particularly sparse in the literature. CHOP and CVAD-based therapies are the most widely used regimens (Liu et al. 2011). Current clinical cases, treatment regimens and outcomes are reported in Table 1.

CNS surveillance should be routinely employed in the management of patients with PBL, particularly as disease progression is widespread and typically involves the CNS (Cha et al. 2010; Ramalingam et al. 2008). Our patient was treated with 6 cycles of hyper-CVAD, CNS chemo-prophylaxis and radiation therapy, with excellent results. Nguyen et al. describe a patient with nasal cavity PBL treated with three monthly courses of hyper-CVAD chemotherapy with CNS prophylaxis [Table 1) using intrathecal methotrexate with each cycle and consolidative locoregional radiation therapy. Biopsy-confirmed (maxillary sinus) complete remission (Nguyen et al. 2003) was achieved.

As presently, for advanced PBL there is no definitive treatment regimen capable of providing curative results. Autologous or allogeneic stem cell transplantation is a therapeutic option for relapsed or refractory disease. Philip and colleagues (Philip et al. 1995) have shown that high dose chemotherapy and autologous bone marrow transplant may significantly improve overall survival in patients with relapses following failed multi-agent chemotherapy in non-Hodgkin’s lymphoma. Recently, Liu et al. (Liu et al. 2011) reported success utilizing consolidation with hematopoietic stem cell transplant in patients with PBL who attained a first complete remission.

Currently, specific randomized clinical trials supporting stem cell transplantation for PBL are lacking. This fact, coupled with the risk of significant transplantation-related morbidity and mortality, suggests that the transplant approach should be reserved for carefully selected cases of PBL. Until a standardized chemotherapeutic regime is identified, therapy for patients with PBL should be considered on a case-by-case basis (Scheper et al. 2005).

## Consent

Written informed consent was obtained from the patient for publication of this report and any accompanying images.
